# Uncovering New Challenges in Targeting Glycolysis to Treat Th17 Cell-Mediated Autoimmunity

**DOI:** 10.20900/immunometab20210006

**Published:** 2021-01-22

**Authors:** Sarah A. Mosure, Laura A. Solt

**Affiliations:** 1 Department of Immunology and Microbiology, The Scripps Research Institute, Jupiter, FL 33458, USA; 2 Department of Integrative Structural and Computational Biology, The Scripps Research Institute, Jupiter, FL 33458, USA; 3 Skaggs Graduate School of Chemical and Biological Sciences, The Scripps Research Institute, Jupiter, FL 33458, USA; 4 Department of Molecular Medicine, The Scripps Research Institute, Jupiter, FL 33458, USA

**Keywords:** Th17 cell, multiple sclerosis, metabolism, glycolysis, pyruvate kinase, inflammation

## Abstract

Targeting glycolysis in T helper 17 (Th17) cells presents an attractive opportunity to treat Th17 cell-mediated autoimmune diseases such as multiple sclerosis (MS). Pyruvate kinase isoform 2 (PKM2) is a glycolytic enzyme expressed in T cells infiltrating the central nervous system in a mouse model of MS, suggesting PKM2 modulation could provide a new avenue for MS therapeutics. In a recent article in *Science Signaling*, Seki et al. show that pharmacological modulation of PKM2 alters but does not ameliorate disease in a mouse model of MS. These results warrant further consideration of PKM2 modulators to treat Th17 cell-mediated autoimmunity.

In multiple sclerosis (MS), autoreactive T cells infiltrate the central nervous system and mount a damaging immune response against myelin, leading to severely debilitating neurological symptoms [1,2]. A central role for T helper 17 (Th17) cells, a subset of CD4^+^ T cells, in MS pathogenesis is supported by a combination of human genetic and clinical evidence, as well as data from the experimental autoimmune encephalomyelitis (EAE) mouse model of MS [1,3]. Th17 cells rely on a coordinated network of transcription factors to regulate development and function [4,5]. Multiple lines of evidence have demonstrated that therapeutically targeting many of these transcription factors is feasible and effective, supporting the rationale for targeting Th17 cells to treat MS [6–8].

Recent work has focused on identifying features unique to pathogenic vs homeostatic Th17 cells that could be exploited for therapeutic benefit [9]. One such characteristic is the altered metabolic profile of pathogenic Th17 cells [10]. Driven by key regulators of aerobic glycolysis, including mTORC, pathogenic Th17 cells exhibit increased glycolysis, which may drive inflammation by facilitating elevated protein, lipid, and nucleic acid synthesis necessary for increased proliferation and pro-inflammatory cytokine production [11,12]. These data suggest that therapeutics targeting glycolytic pathways could be effective treatments for Th17 cell-mediated autoimmune diseases [7]. Indeed, genetic ablation or pharmacological inhibition of glycolytic enzymes protects against disease in EAE [7,13].

Specifically targeting glycolysis in pathogenic Th17 cells while minimizing effects on other cell types [9] is challenging given glycolysis is essential in most cells [14]. Excitingly, recent evidence suggests the glycolytic enzyme pyruvate kinase could provide a means to selectively target glycolysis in T cells [15]. Alternative splicing dictates cell type-specific expression of isoforms including pyruvate kinase isoform 1 (PKM1) and pyruvate kinase isoform 2 (PKM2) [16]. PKM1 exhibits robust enzymatic activity to provide pyruvate necessary for oxidative phosphorylation and ATP production in terminally differentiated cells [17]. In contrast, PKM2 is enzymatically less active and instead functions as a transcriptional coactivator, allowing for shunting of glycolytic intermediates to anabolic pathways [18,19]. Notably, T cells exclusively express PKM2, suggesting pharmacological modulation of PKM2 could provide the means to target T cell glycolysis with greater selectivity [15].

In a recent article in *Science Signaling*, Seki et al. explored the effect of the PKM2 activators, TEPP-46 and DASA-58, on Th17 cell activity in vitro and in vivo [20]. TEPP-46 and DASA-58 have been designed to enhance the enzymatic activity of PKM2 so that it functions more like PKM1 [21,22]. Pharmacological PKM2 activation should divert metabolic intermediates toward catabolic processes and ATP production in lieu of anabolic processes and proliferation [23], which would be expected to inhibit pro-inflammatory T cell activity.

Consistent with previous reports [15], the authors found PKM2, but not PKM1, is upregulated following T cell receptor (TCR) activation in CD4^+^ T cells cultured under Th17-skewing conditions. In macrophages, which also exclusively express PKM2, TEPP-46 and DASA-58 treatment inhibited a pro-inflammatory phenotype [24]. Similarly, PKM2 activator treatment was previously shown to inhibit pro-inflammatory cytokine production by Th17 cells in vitro and in vivo [15]. These data align with that presented by Seki et al., in which PKM2 activators inhibited production of the signature pro-inflammatory Th17 cell cytokine IL-17A.

Although the inhibition of IL-17A is congruent with previous studies, Seki et al. uniquely observed increased pro-inflammatory cytokines IFNγ and GM-CSF and no significant effects on glycolysis or oxygen consumption with PKM2 activator treatment. While this discrepancy could be due to the earlier timepoint analyzed, another explanation is that Seki et al. used total CD4^+^ T cells, a more heterogenous mixture of naïve and memory cells, while the previous study enriched for naïve CD4^+^ T cells. This is significant in light of data showing PKM2 activators inhibit TCR activation [15]. Memory cells may exhibit fewer defects in activation in the presence of PKM2 activators, resulting in a different cytokine and metabolic response. Future studies directly comparing memory vs naïve cell responses to PKM2 activator treatment are needed to confirm this possibility. This issue will be critical to address considering therapeutics for autoimmune diseases are administered after disease onset (i.e., affecting memory cells), not as preventative treatments (i.e., affecting naïve cells).

Data by Seki et al. also raises concerns regarding the specificity of PKM2 activators. PKM2 activator treatment of PKM2 conditional knockout (KO) T cells produced a similar increase in GM-CSF and decrease in IL-17A expression as treatment of WT T cells. Further investigation revealed compensatory upregulation of PKM1 in PKM2 KO Th17 cells. Although PKM2 activators were previously shown to exhibit high selectively for PKM2 vs PKM1 [21], a clickable TEPP-46 analogue pulled down both PKM2 and PKM1 from Jurkat T cell lysates, challenging the notion that TEPP-46 activity is specific to PKM2 (Figure 1). These results suggest small molecules thought to be PKM2-specific can also exhibit off-target effects on PKM1 that may operate independently of PKM1 enzymatic activity. This finding could explain why, in a separate, study, the use of a PKM2 inhibitor, shikonin, significantly reduced Th17 cell differentiation and the development of EAE [25]. Compounds affecting the activity of both PKM1 and PKM2 (TEPP-46) could be expected to have different readouts than a compound that is reported to have activity at PKM2 only (shikonin) [26]. Finally, it is interesting to note that the compensatory upregulation of PKM1 in PKM2 KO Th17 cells was also reported in NK cells [27]. These data beg the question: are there truly compensatory mechanisms at play in the absence of PKM2, or is the deletion of the PKM2 exon 9 resulting in artefactual expression of the PKM1-specific exon 10? These concerns require further investigation, including different strategies to assess PKM2 activity in Th17 cells.

Concerns over lack of specificity raised by the PKM2 KO mice are significant in light of the experimental procedures previously used to show PKM2 activators inhibit Th17 cell-mediated autoimmune disease in vivo. In the previous study, which used an active EAE induction model, mice were treated with TEPP-46 post-immunization to determine the effect on disease progression [15]. This method contrasts with the passive EAE disease induction procedure used by Seki et al., in which myelin oligodendrocyte glycoprotein (MOG)-specific TCR transgenic 2D2 cells were cultured under Th17-skewing conditions in the presence of TEPP-46 before transferring to *Rag1*^*−/−*^ recipients. The latter method ensures effects on disease progression are exclusive to modulation of Th17 cells, while in the former system, it cannot be concluded whether amelioration of disease is specific to modulation of Th17 cells or any other cell type, since drug exposure is systemic.

Because the passive EAE model used by Seki et al. confers greater specificity, the results better represent how TEPP-46 affects Th17 cell activity in a model of MS. Thus, it is concerning that TEPP-46 treatment increased T cell homing to the brain and atypical EAE, associated with ataxia, rather than inhibiting Th17 cell-mediated inflammation. It is also problematic that TEPP-46 inhibited development of T regulatory cells by interfering with TGFβ signaling, since T regulatory cells are critical for repressing inflammation.

While the findings by Seki et al. suggest the existing PKM2 pharmacological activators (e.g., TEPP-46) may not be as effective as previously indicated, this does not necessarily mean PKM2 is not a viable target for the treatment of Th17 cell-mediated autoimmunity. It remains unclear whether many of the effects of TEPP-46, including those within Th17 cells, are specific to PKM2 modulation, and evidence suggests pharmacological inhibition of PKM2 may prove more effective than activation [25]. Overall, further studies using genetic modulation of PKM2 (e.g., CRISPR to generate activating PKM2 mutants) are needed to better understand mechanisms underlying PKM2 activity in Th17 cells. It will also be important to evaluate whether reported effects translate to human Th17 cell development. Collectively, these studies should help inform the design of PKM2 modulators with better therapeutic efficacy.

## Figures and Tables

**Figure 1.**
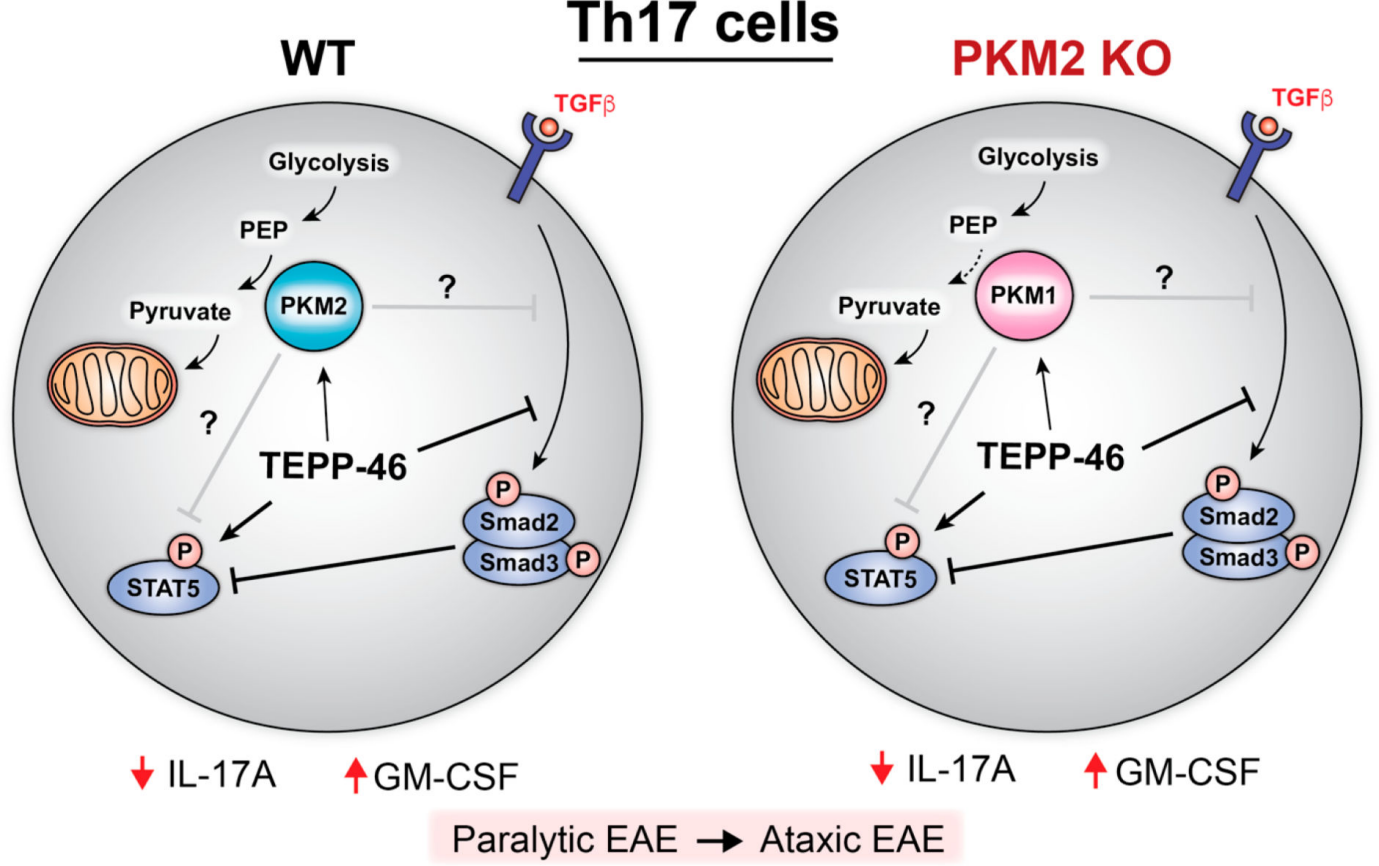
Effects of TEPP-46 treatment on WT and PKM2 KO Th17 cells based on data from Seki et al. TEPP-46 is thought to enhance conversion of phosphoenolpyruvate (PEP) to pyruvate by PKM2 in the final step of glycolysis, but neither Th17 cell glycolysis nor oxidative phosphorylation is affected by TEPP-46 treatment. TEPP-46 increases STAT5 and decreases Smad2 phosphorylation; these effects are correlated with less IL-17A and more GM-CSF in both WT and PKM2-KO treated cells. It remains unclear whether these effects are directly due to PKM activation.

## ABBREVIATIONS

Th17: T helper 17
MS: multiple sclerosis
EAE: experimental autoimmune encephalomyelitis
PKM2: pyruvate kinase isoform 2
TCR: T cell receptor
KO: knockout
MOG: myelin oligodendrocyte glycoprotein
